# Importance of Creatinine Generation Rate in Estimating Glomerular Filtration Rate

**DOI:** 10.7759/cureus.80441

**Published:** 2025-03-11

**Authors:** Minhtri K Nguyen, Dhiresh Bandaru

**Affiliations:** 1 Medicine, Ronald Reagan University of California Los Angeles Medical Center, Los Angeles, USA

**Keywords:** creatinine (creat), creatinine generation rate, e-gfr, glomerular filtration rate (gfr), renal function

## Abstract

Assessment of glomerular filtration rate (GFR) is commonly based on serum creatinine levels. It is well-established that, for a given GFR, the mean serum creatinine level is higher in Black individuals than in non-Black individuals. Failure to account for this difference in creatinine-based GFR estimation equations leads to inaccuracies in GFR estimates for Black individuals. This higher mean serum creatinine level in Black individuals has been attributed to greater muscle mass, though this explanation remains debated. In this article, we provide mathematical proof showing that the higher mean serum creatinine level in Black individuals reflects the “net” creatinine generation rate after accounting for renal tubular secretion of creatinine and extrarenal creatinine elimination. To accurately estimate GFR, this “net” creatinine generation rate must be considered. By rescaling serum creatinine to account for the differences in creatinine production rates between Black and non-Black populations, the population-specific creatinine-based European Kidney Function Consortium (EKFC) equation provides a more accurate GFR estimate than the current Chronic Kidney Disease Epidemiology Collaboration (CKD-EPI 2021) equation.

## Editorial

Introduction

At any given GFR, the mean serum creatinine level is higher in Black individuals compared to non-Black individuals [1]. This difference has been attributed to biological variations in non-GFR determinants, such as muscle mass or creatinine handling [1,2]. Recently, Delanaye et al. developed a new European Kidney Function Consortium (EKFC) creatinine-based equation, which accounts for varying creatinine generation rates in Black and non-Black individuals in US cohorts [3]. The equation uses a rescaled serum creatinine (sCr/Q), where sCr represents the individual creatinine level and Q represents the average creatinine value for healthy persons of the same population. Delanaye et al. proposed that this rescaling, using population-specific Q values, accounts for the different creatinine production rates in Black and non-Black populations. In this article, a mathematical proof is derived to confirm the validity of this approach. 

Non-GFR determinants of the serum creatinine 

Serum creatinine is a by-product of creatine breakdown in skeletal muscle and from dietary intake of cooked meat [2]. As a result, creatinine generation varies based on factors such as muscle mass (e.g., malnutrition, muscle wasting, amputations, age, sex, and race) and dietary intake (e.g., vegetarian diet, ingestion of cooked meat, and creatine supplements). Serum creatinine levels are also influenced by tubular secretion of creatinine and extrarenal creatinine elimination by intestinal bacterial creatininase activity [2]. Proximal tubular creatinine secretion and extrarenal creatinine elimination are enhanced as the GFR declines [2]. As creatinine is increasingly eliminated through the extrarenal pathway with worsening renal function, this can lead to an overestimation of GFR by creatinine-based estimated GFR formulas. Renal tubular creatinine secretion is also increased in sickle cell disease and decreased by certain drugs, independent of GFR [2]. In individuals with sickle cell disease, enhanced proximal tubular function leads to increased creatinine secretion, which may result in an overestimation of true GFR using creatinine-based formulas. Conversely, drug-induced inhibition of renal tubular creatinine secretion can increase serum creatinine without affecting true GFR, leading to an underestimation of GFR. Similarly, drug-induced myopathy can elevate serum creatinine due to increased creatinine production. In cases of drug-induced rhabdomyolysis, the rise in serum creatinine may be caused by both increased creatinine production and a decrease in GFR due to acute kidney injury. Lastly, errors in serum creatinine measurement can occur due to interference from various substances affecting the creatinine assay [2].

Discussion

**Figure 1:**
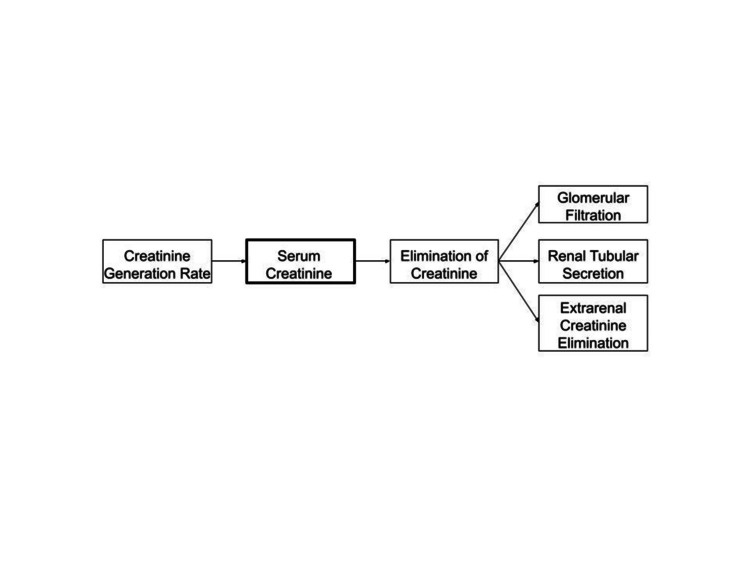
GFR and non-GFR determinants of the serum creatinine Serum creatinine has GFR and non-GFR determinants. In the steady state, the creatinine generation rate is equal to the elimination of creatinine by glomerular filtration, renal tubular secretion, and extrarenal creatinine elimination. Since there are non-GFR determinants, the “net” creatinine generation rate after accounting for renal tubular secretion of creatinine and extrarenal creatinine elimination must be accounted for in order to accurately estimate GFR when using any creatinine-based GFR estimating equation. Image Credit: Authors' original creation.

To accurately estimate the GFR, creatinine-based GFR estimating equations must account for the non-GFR determinants of the serum creatinine. In the steady state, the creatinine generation rate is equal to the elimination of creatinine by glomerular filtration, renal tubular secretion, and extrarenal creatinine elimination (Figure 1).

Based on Figure 1, we get: Creatinine generation rate = glomerular filtration of creatinine + renal tubular secretion of creatinine + extrarenal creatinine elimination (Eq. 1).

Since glomerular filtration of creatinine is GFR x (serum creatinine) (Eq. 2), the creatinine generation rate becomes: Creatinine generation rate = GFR x (serum creatinine) + renal tubular secretion of creatinine + extrarenal creatinine elimination (Eq. 3).

Rearrangement of this equation yields: GFR = (creatinine generation rate - renal tubular secretion of creatinine − extrarenal creatinine elimination) ÷ (serum creatinine) (Eq. 4).

We refer to the difference of “creatinine generation rate - renal tubular secretion of creatinine − extrarenal creatinine elimination” as the “net” creatinine generation rate after accounting for renal tubular secretion of creatinine and extrarenal creatinine elimination.

It is well known that at any given measured GFR, the mean serum creatinine level is higher in Black individuals compared to non-Black individuals [1]. Therefore, according to Eq. 4, since the mean serum creatinine level (denominator) is higher in Black individuals at any given GFR, then the “net” creatinine generation rate (numerator) after accounting for renal tubular secretion of creatinine and extrarenal creatinine elimination must be higher in Black individuals compared to non-Black individuals to yield the same measured GFR. 

Similarly, at any given serum creatinine level, the measured GFR is higher in Black individuals compared to non-Black individuals [1]. 

Rearrangement of Eq. 4 yields: serum creatinine = (creatinine generation rate - renal tubular secretion of creatinine − extrarenal creatinine elimination) ÷ GFR (Eq. 5).

Since the measured GFR (denominator) is higher in Black individuals at any given serum creatinine, the “net” creatinine generation rate (numerator) after accounting for renal tubular secretion of creatinine and extrarenal creatinine elimination must be higher in Black individuals compared to non-Black individuals to maintain the same serum creatinine level.

To accurately estimate GFR across different populations, the “net” creatinine generation rate after accounting for tubular secretion of creatinine and extrarenal creatinine elimination must be considered. Recently, Delanaye et al. developed a new European Kidney Function Consortium (EKFC) creatinine-based equation that accounts for varying creatinine generation rates in US cohorts [3]. This equation aims to make GFR estimating equations applicable across populations with different creatinine generation rates by using rescaled serum creatinine (sCr/Q), where sCr represents the individual creatinine level and Q represents the average creatinine value in healthy persons of the same population. Based on Eq. 5, the population-specific Q-value is a surrogate for the “net” creatinine generation rate after accounting for tubular secretion of creatinine and extrarenal creatinine elimination in a given population. By accounting for the different creatinine generation rates in US cohorts, the population-specific, creatinine-based EFKC equation showed no statistical median bias (0.14, 95% confidence interval -0.07, 0.35 mL/min/1.73 m^2^). This equation is more accurate than the current Chronic Kidney Disease Epidemiology Collaboration (CKD-EPI 2021) equation (median bias 1.22, 95% confidence interval 0.99, 1.47 mL/min/1.73 m^2^) [3]. The percentage of estimated GFR within 30% of measured GFR was 79.2% (78.5%; 79.9%) for the CKD-EPI 2021 equation but improved for the population-specific, creatinine-based EKFC equation (81.1% (80.5%; 81.8%)). By providing a more accurate estimate of GFR, the population-specific, creatinine-based EKFC equation will improve the accuracy of CKD staging and reduce misclassification, thereby leading to better therapeutic decisions. Future studies will be needed to study the impact of CKD staging in the US population. It should be noted that the EKFC equations have been developed and validated in predominantly European and North American populations [3,4]. The population-specific, creatinine-based EKFC equation has also been studied in a small African cohort of 508 Black patients, and it has also been shown to be more accurate than the CKD-EPI equations [4].

As with any creatinine-based GFR estimating equation, the population-specific, creatinine-based EKFC equation may not be applicable for any given individual since the Q-value represents the average creatinine value in healthy persons of the same population and may not reflect differences in the “net” creatinine generation rate at the individual level. In contrast, cystatin C-based GFR estimating equations are not dependent on the “net” creatinine generation rate. For instance, the 2012 CKD-EPI cystatin C equation does not require a race variable, whereas the addition of creatinine as a variable in the 2012 CKD-EPI creatinine-cystatin C equation requires a race variable for greater statistical accuracy [5]. It has also been shown that GFR can be estimated based on the EKFC cystatin C-based equation using a rescaled cystatin C level. Similar to the 2012 CKD-EPI cystatin C equation, the EKFC cystatin C-based equation does not require a correction factor for race because the rescaled cystatin C levels were similar between Black and White individuals [4]. However, GFR is best estimated by the 2012 CKD-EPI creatinine-cystatin C equation and EKFC creatinine-cystatin C-based equation. Therefore, the inclusion of the creatinine variable in the combined creatinine-cystatin C-based equations requires consideration of the “net” creatinine generation rate to accurately estimate GFR.

In summary, accurately estimating GFR requires accounting for the "net" creatinine generation rate, which considers renal tubular secretion and extrarenal creatinine elimination. The use of rescaled serum creatinine (sCr/Q) is a logical approach to make GFR estimating equations applicable across populations with different "net" creatinine generation rates.

## References

[1] Levey AS, Bosch JP, Lewis JB, Greene T, Rogers N, Roth D (1999). A more accurate method to estimate glomerular filtration rate from serum creatinine: a new prediction equation. Ann Intern Med.

[2] Alaini A, Malhotra D, Rondon-Berrios H (2017). Establishing the presence or absence of chronic kidney disease: uses and limitations of formulas estimating the glomerular filtration rate. World J Methodol.

[3] Delanaye P, Rule AD, Schaeffner E (2024). Performance of the European Kidney Function Consortium (EKFC) creatinine-based equation in United States cohorts. Kidney Int.

[4] Pottel H, Björk J, Rule AD (2023). Cystatin C-based equation to estimate GFR without the inclusion of race and sex. N Engl J Med.

[5] Inker LA, Schmid CH, Tighiouart H (2012). Estimating glomerular filtration rate from serum creatinine and cystatin C. N Engl J Med.

